# Giok the Alien: An AR-Based Integrated System for the Empowerment of Problem-Solving, Pragmatic, and Social Skills in Pre-School Children

**DOI:** 10.3390/s18072368

**Published:** 2018-07-21

**Authors:** Maria Luisa Lorusso, Marisa Giorgetti, Simona Travellini, Luca Greci, Andrea Zangiacomi, Marta Mondellini, Marco Sacco, Gianluigi Reni

**Affiliations:** 1 Scientific Institute IRCCS E. Medea, Via don Luigi Monza 20, 23842 Bosisio Parini (LC), Italy; marialuisa.lorusso@bp.lnf.it (M.L.L.); marisa.giorgetti@unicatt.it (M.G.); simona.travellini@bp.lnf.it (S.T.); gianluigi.reni@bp.lnf.it (G.R.); 2 Istituto di Tecnologie Industriali e Automazione-Consiglio Nazionale delle Ricerche Via Corti 12, 20133 Milano, Italy; andrea.zangiacomi@itia.cnr.it (A.Z.); marta.mondellini@gmail.com (M.M.); marco.sacco@itia.cnr.it (M.S.)

**Keywords:** augmented reality, children, cooperative games, interactive learning environments, pragmatic skills, communication disorders, empowerment

## Abstract

The use of technology for educational purposes is a consolidated reality, and many new tools are constantly being devised and offered for use with both normally developing children and children with special needs. Nonetheless, a detailed analysis of the processes being stimulated and of the goals being pursued is often lacking or absent. In this work we describe the design, development and preliminary testing of an integrated system which combines the use of smart devices, a physical cube, augmented reality (AR) technology, a smart TV, and a software application especially designed to stimulate cognitive and social functions in pre-school children. The system was tested with three groups of children (25 children in total) during kindergarten activities. The results show that the system is easy to understand, elicits high levels of participation and social interaction, favors strategic behaviors, and can be used by the children with limited need of instruction and support by the adult. The implications for empowerment in typically developing children and the possibilities for use with children who have specific impairments in social communication are discussed.

## 1. Introduction

Modern technology and Virtual or Augmented Reality offer unlimited opportunities to stimulate specific skills and functions in an entertaining, engaging fashion [1,2].

Virtual Reality (VR), in general terms, is a technology that allows for replacing the real world with a synthetic one, creating computer-generated virtual environments where users can experience and interact in a similar way as they would do in real life [3]. Augmented reality (AR) overlays virtual objects on the real-world environment allowing the users to see and interact with the real world while digital content is added to it. This makes them perfect instruments for gaming purposes, not only with the aim to entertain, but also to train through gaming, what is usually referred to as edutainment or serious gaming. Moreover, as underlined in [4], “the educational values of AR are not solely based on the use of technologies but closely related to how AR is designed, implemented, and integrated into formal and informal learning settings”. The general psycho-educational framework is that of neuroconstructivism, where cognitive development occurs not through passive absorption of environmental information, but rather through the pro-activity of the child in exploring, manipulating and interacting with his/her environment [5]. More specifically, according to the theory of social constructivism [6], it is essential to construct a learning environment in which students can collaborate and participate actively, experiment, share and develop ideas, use language to reason, and plan and review one’s actions.

The conceptual framework known as TEL—Technology-Enhanced Learning—brings this principle further, suggesting that technology can help the construction of new rules through the interaction with data in the learning environment. Comprehension would be achieved through activity and technology can provide the tools and contexts for activity to take place. According to [7], games encourage exploring, exchange of ideas, communication, and decision-making. The players become active builders of new knowledge. Playing a game requires the ability to interpret images, sounds, and actions, as well as written words [8]. It depends on the player’s ability to understand and learn the game’s multimodal features, what Gee describes as its “internal design grammar” [9] by probing the virtual world of the game, forming hypotheses about it, re-probing it in light of those hypotheses in mind, and then, based on feedback from that virtual world, accepting or re-thinking the hypotheses. This process is at the base of the scientific method [9] but it is also, more generally, the basic structure of problem-solving. Although empowerment of problem-solving abilities is more often referred to as a focus and target for adolescents and adults, it has been shown how important it is to stimulate problem-solving skills and critical thinking very early in life, involving perception, motor skills, and cognition at the same time [10,11]. It was shown with preschool children that playful, exploratory learning produces more creative and flexible use of materials than does explicit training from an adult [12]. Moreover, preschoolers enjoy problem solving. They get more involved in playing when actions produce ambiguous than unambiguous results [13].

In this line of reasoning, Prensky [14] suggests that the modern type of game that particularly challenges problem-solving abilities is video games, stimulating skills, such as decision-making, data handling, multi-tasking, and information processing. This can involve not only the single individual, but a whole group of individuals collaborating to solve the given problem, in what is called “distributed cognition” and finds its more widely known model in computer-supported collaborative learning (CSCL) [15]. It should be observed that the main paradigms of computer-assisted learning place even more emphasis on the group dimension and on collaboration than on the individual student who learns and thinks. Even if it is not denied that individuals can think and learn on their own, the accent is on situations of collaborative activity where processes of learning and cognition take place at the group level [16]. What is referred to as “problem-solving skills” at the cognitive-behavioral level is basically described as Executive Functions at the neuropsychological level, i.e., taking into consideration specific processes and mechanisms resting on the neurobiological substrate and changing along its development. In Diamond’s [17] description, a series of functions including working memory, inhibition, and cognitive flexibility (shifting) all concur to the development of EF, though each following its own developmental trajectory. Nonetheless, an overarching ability to coordinate EF components shows a dramatic improvement in two developmental periods, the first one occurring in the last half of the first year and the second one spanning from 3–6 years of age [18]. As described by Garon et al. [19], EF is the ability to overcome automatic, prepotent behavior despite the pull of previous experience [18,20]. The ability to deal with conflict during information processing is considered a critical EF development during the preschool period. Additionally, Posner and Rothbart [21] highlight the role of conflict resolution in the development of EF and of attention more in general. Overall, studies converge on showing that the gains resulting from training single components of EFs in children can be hardly generalized and transferred to other activities, and do not necessarily benefit problem-solving abilities [18]. Multimodal approaches, also including curriculum-based programs, seem to be more effective and produce gains extending to socio-emotional regulation [22]. Thus, directly training problem-solving skills in real-life-related situations appears to be the best choice.

This is especially crucial when addressing the needs of children, who are less motivated to devote time and effort to their specific weaknesses even when this would be of great advantage. Games, serious games, or edugames represent a unique resource to pursue rehabilitative goals without necessarily involving the children in long and effortful therapy sessions, but rather exploiting their own interests. Some children, namely children with autistic spectrum disorders (ASD) [23] or with social (pragmatic) communication disorders [24], specifically lack the ability to efficiently use communication strategies and skills to engage in social interactions with their peers. They may present a poor speech repertoire, repetitive language, gaze avoidance, withdrawal, disorientation, and echolalia. These conditions may interfere with daily life, preventing real integration [25] and inclusion within educational and community settings, with large negative impacts on quality of life [26]. They either show little interest in these interactions, or simply do not know how to initiate and maintain them in a way that is rewarding for both themselves and their interlocutor. In the past few years, therefore, programmers in the Android and iOS communities have come up with various applications for education, development, communication, and rehabilitation for children with autism and communication disorders [27]. Such applications may rely on high technology devices, such as computers, tablets, and iPads. Interventions based on Assistive Technology (AT) seem to be able to improve self-determination and independence of children with ASD by promoting an active role, with beneficial effects on their social image, desirability, and status [28,29]. With regard to communication skills, AT-based interventions increase the opportunity of requesting preferred items, ignoring non-preferred and/or neutral activities, beginning conversational interactions. These tools are exemplars of the constructivist concept of learning, they empower children to plan and monitor their own learning process, encourage generalization of the learned principles to new situations, and foster collaborative learning activities [30,31,32].

Storytelling is one of the activities that have been shown to be particularly helpful in the rehabilitation of children with social communication disorders. Indeed, storytelling is essential for children’s linguistic and cognitive development [33]. Gray [34] developed Social Stories as intervention tools for children with autism. Social Stories are short scenarios especially written for autistic individuals that should help them understand and behave appropriately in social situations [35]. A few researchers have proposed incorporating augmented reality into storytelling systems [36]. Montemayor et al. [37], for example, created a physical interactive environment to enrich the storytelling system in elementary and high school education. It is only very recently that such systems have been applied in preschool settings. A storytelling system within a computer-supported instructional tool is a typical engaging learning environment that can be used to support constructive learning in an educational setting, but information on how this works, especially with very young children, is still sparse [38].

Recently, increasing attention has been attracted by tangible user interfaces (TUIs), a new interface type bridging the digital and physical worlds [39]. TUIs aim to enhance the efficiency with which people interact with digital information, drawing upon the ability to interact with the real non-digital world. Studies in this area are still few, however, this approach seems particularly promising especially referring to very young children or children with special needs, who may have difficulties bridging the gap between digital and real-world representations.

The application presented here is part of a set of ICT-based educational tools for pre-school children that were developed and preliminarily tested to provide effective stimulation of a wide range of cognitive and social abilities in children with typical and atypical development. The app specifically addresses pragmatic skills and simple rules of social interaction by providing a digital context and a funny character (Giok the alien) whose behavior is decided by the child through serial multiple-choice options which will or will not lead to a positive outcome. The interaction with the cube allows the user to select the scenario they want to play among the different options available, to start playing the chosen scenario and take decisions about what to do when they have to face the problem-solving activities. Thus, through simulated problem-solving instances and identification with the funny hero of the story, the child faces real-life situations where social skills are needed and can experiment with different strategies and solutions in order to find the best response leading to a satisfactory solution of the problem.

To increase the learning outcomes and the level of engagement of the users each problem-solving activity and the related options have been enriched with sounds and animations.

The app has been tested in a mainstream kindergarten situation with a group of preschoolers representing an unselected sample of the school population, including children with varying levels of ability, in an inclusive perspective where activities for empowerment are proposed to the whole group. The implication is that each child will face the new activity with his own background of skills and difficulties, and that empowerment will occur at the group level for collaboration, interaction, communication, and collective strategies; at the individual level, each child will be stimulated and challenged by the task and by the group, in a non-intrusive and non-anxiety-inducing way, where support is constantly provided by adults and peers in interaction. 

An ad-hoc constructed observation grid was used to record the children’s reactions and behaviors during the activity. Indeed, it has recently been increasingly argued that the assessment of development processes and learning during preschool should be done primarily through systematic observation in the natural learning context (Early Head Start National Resource Center, 2013; [40]). The grid was constructed so as to be able to highlight play and social behaviors, as well as problem-solving attitudes and skills (in a similar way as other instruments created to describe preschoolers’ behavior during play with toys [41]), and their changes through session time.

As highlighted by a recent systematic review of the literature [28] the main advantages of AR in the educational context are learning gains, motivation, interaction, and collaboration. Indeed, formal learning is not envisaged at the kindergarten level, however, problem-solving skills and executive functions, such as planning, monitoring, and revising one’s actions are crucial abilities that belong to the educational goals at this stage.

Therefore, the aim of the present study was to observe the impact of the application on both cognitive and social processes, including interaction, collaboration, participation, conflict management, and strategic behavior, but also to give some insights on accessibility/usability of the app, as emerging from the children’s observed behaviors (showing understanding of the functional principles of the application, asking questions about the device and requesting help from the adult observers). Moreover, we wanted to describe how this impact is modified through experience and familiarity with the device. Indeed, it was expected that comprehension of the game structure and function, as well as the strategic approach, could increase through time, and that the ability to cooperate and positively interact would also increase as a consequence of growing confidence and understanding. Further, we were interested in testing whether, by contrast, the interest elicited by the game would decrease over time (much like a “novelty effect”), and whether negative social mechanisms related to intra-peer conflict could arise as a consequence of greater mastery of the activity and increased self-confidence. 

The present contribution describes the observed effects at the group level as a first step and as a pre-requisite in the validation of the instrument and preceding the investigation of its effects at the individual level.

In the next section, the task and the structure of the “Giok the alien” application will be illustrated, with insights on the technical equipment needed, on how it works, and on software development. Then, the testing phase of the application will be described, as it was carried out with the children of an Italian kindergarten. The results of a systematic observation carried out by field experimenters and independent observers will be outlined and discussed. More specifically, the results of the experimentation will be detailed through aggregated scores obtained from the grids filled in by observers on the macro areas of interest: the game, the interaction with peers, and the requests for clarification and help. Finally, the results will be commented, focusing on possible, appropriate uses of the application, on the advantages and disadvantages it can bring for preschool education in a kindergarten context and on possible future developments.

## 2. Materials and Methods

### 2.1. The Task

The app “Giok the alien” is built to stimulate problem solving skills in various contexts, both for typically developing children and for those who have specific impairments in social communication. Different scenarios have been designed. In each scenario, the child is faced with more or less usual problem situations that require a choice between different options in order to progress on the game path.

Three scenarios have been realized to stimulate problem solving abilities, each involving different skills and functions: “Playground”, focusing on social and pragmatic skills and on theory of mind; “Cocoa plantation”, focusing on the factors underlying a growth process (from the cocoa bean to the cup of hot chocolate); “World cuisine”, encouraging stimulus association, semantic enrichment, and pursuing additional objectives related to food education. In this paper only data regarding the “Playground” scenario will be considered.

**Figure 1:**
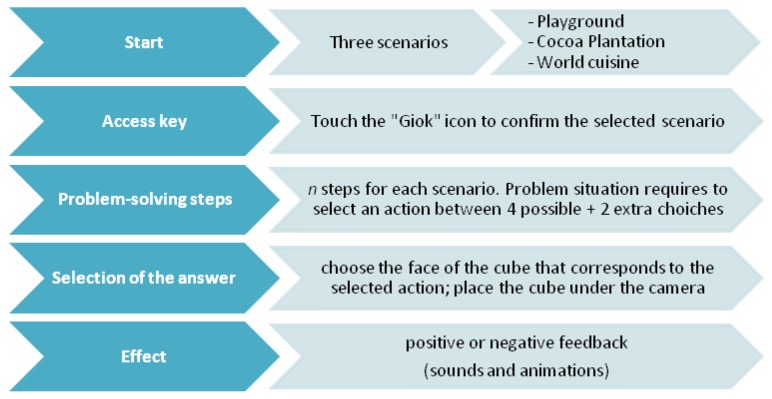
The game flow in “Giok the Alien”.

The game flow with “Giok the Alien” is shown in Figure 1.

Each step of the game (n-step presented according to the different scenarios) provides the animated presentation of the problem situation, with the subsequent display of four response options in the form of icons. The selection of an action results in the continuation of the path, its interruption, the permanence in the problem situation, or the opening of an intermediate problem.

Specifically, the proposed alternatives aim to place the child in situations that are not necessarily dichotomous (right/wrong response), but functional to stimulate logical reasoning, strategic thinking, and socially-shared responses. The four possible choices represent: the correct choice, the incorrect choice, an irrelevant choice and a partially correct choice, which introduces an intermediate response request with two further alternatives.

Each choice has a different consequence: (1) the correct choice allows players to move to the next step; (2) the incorrect choice sends back to the first step of the scenario; (3) the irrelevant choice implies no changes in the current situation, but leaves only three possible answers available; (4) the partially correct choice leads to an intermediate problem to be solved: at this point only two possible solutions are available, a correct one and an incorrect one. After a choice is made, an animation shows the effects of that choice with respect to the proposed problem.

**Figure 2:**
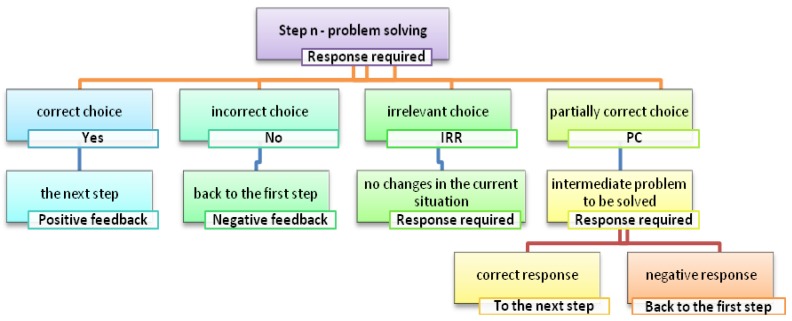
Structure of n-Step in “Giok the Alien”.

The structure described in Figure 2 has been used to define the detailed storyboards useful for the development of the application and the creation of graphic animations.

Furthermore, during the design phase the operating guidelines for the use of the application and the development of the game phases were described. Specifically, the first level of the game includes four alternative choices (Yes, No, IRR—irrelevant, PC—partially correct); the second level of the game opens only in case of selection of the “PC” choice, that is, partially correct.

The “Giok the Alien” application allows not only individual fruition on a single device (tablet), but also fruition as a group. In fact, through the use of a micro-USB/HDMI connector it is possible to enjoy the display both on a TV/Monitor and on a projector (collective use).

Children can provide the response required both with the touch of the icon on the device screen, and with the use of the physical cube and the recognition of the markers in augmented reality (AR). For this study, data from collective use were considered.

### 2.2. Equipment

The system is composed of: a smart device, optionally a smart TV connected by cable or wirelessly to the smart device for a collaborative gaming session, the AR software application, and a 3D-printed cube with a marker stuck on each face to enable the AR functionalities.

The developed augmented reality App enables the children to face three scenarios which train either social/pragmatic skills or problem-solving skills or both, according to the different tasks addressed in each specific scenario. The three scenarios represent the following social interactive situations: a day at the park (1), grow cocoa (2), and food from the world (3). In the first scenario (1) the children have to face ten situations that can occur during a day spent at the park. In the scenario (2) the children have to help the farmer during all the phases (10) of cocoa production, from seed to harvest, while in scenario (3) they have to recognize which is the local food of a specific country, based on a series of hints and choosing between plausible and implausible suggestions.

During the design of the characters and scenes particular attention has been paid to the graphics, sounds and animations so that they appear easily understandable, fun, and never arouse any kind of fear in the users.

The App can run on all the devices equipped with the Android operation system. For the validation phase a Sony Xperia Z2 (Sony Mobile Communications Inc., Lund, Sweden) tablet has been used. It features a Qualcomm^®^ Snapdragon™ 801 processor with quad custom Krait CPUs and a graphics processing unity Qualcomm^®^ Adreno™ 330 GPU (Qualcomm, San Diego, CA, USA). The RAM is 3 GB. It provides a micro USB port with MHL technology as well as the Miracast wireless display standard for the connection with the smart TV. The tablet’s screen is 10.1 inches with a 1900 × 1200 (FULLHD) resolution.

At the beginning of the session the start game screen appears and the different scenarios available are presented. To play a scenario the user has to show the physical cube to the device camera. The choice of a physical cube, which represents a tangible interface metaphor, typically associated to gaming [42], has been driven by the need to avoid interaction devices like a mouse and keyboard that are less usable by children, in particular by children with impairments [43].

**Figure 3:**
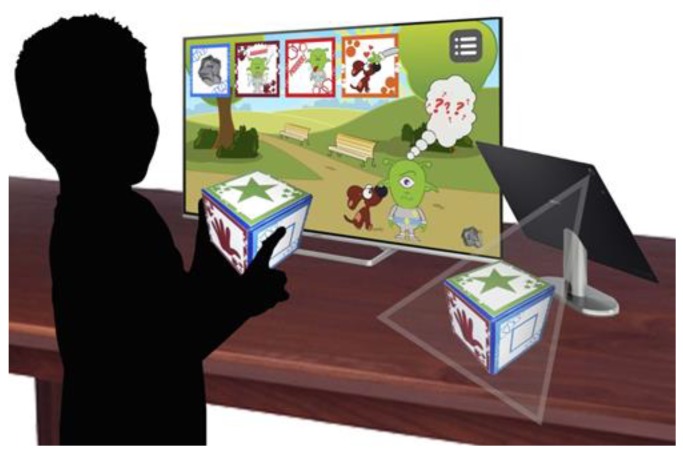
The first scenario (a day at the park).

The matching between the options presented by the application through images and the markers on the physical cube is facilitated by the correspondence between the color and the frame graphic image that is present on the marker. The marker is an image known by the system and an ID is associated to each marker. The choice of the scenario is made by placing the physical cube with the face representing the selected option under the smart device camera (Figure 3).

**Figure 4:**
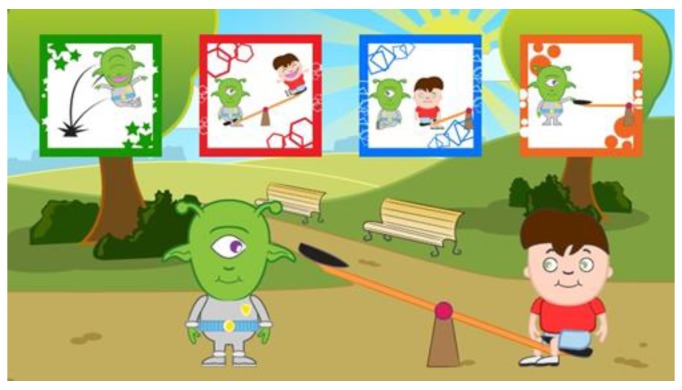
Example of the four options proposed to the user.

When the users try to solve the problem-solving situations, they are asked to select one of the four different available options characterized by related markers (Figure 4). Each marker identifies one of the four possible choices (the correct choice allowing players to move to the next step; the incorrect choice sending back to the first step; the irrelevant choice producing no changes; the partially correct choice leading to an intermediate problem to be solved). Each choice starts the execution of an animation showing the effects of the selection with respect to the problem situation.

When the scenario has been completed, the application gives feedback to the user to highlight the positive result of the choices made and brings back to the home screen. A short example from the “Playground” scenario is provided in the Supplementary Materials.

**Figure 5:**
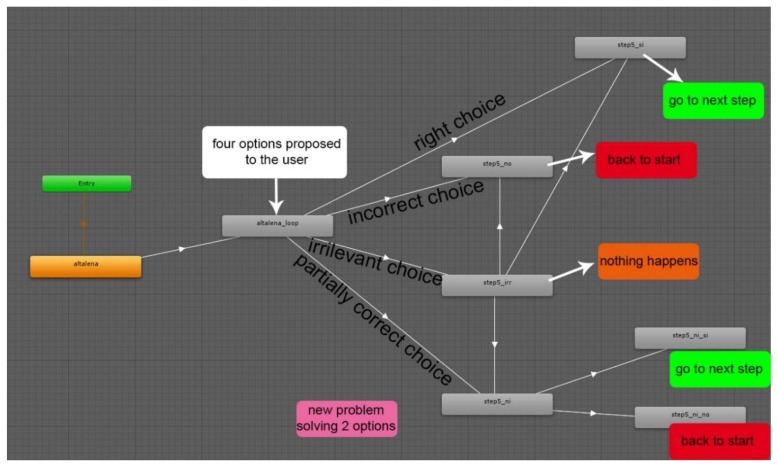
Schematic diagram of a single step.

The application was developed using Unity 3D (Unity Technologies, San Francisco, CA, USA) [44,45] both for writing the code to manage the behavior of the application and for the development and management of the animations. The augmented reality features have been taken from the Vuforia library (PTC Inc, Needham, MA, USA). All the images used to generate the animation have been created using Adobe Illustrator (Adobe Systems Incorporated, San Jose, CA, USA), while the animation has been realized using the animation tool provided by Unity 3D. The animation management has been implemented through the Unity 3D state machine module (Figure 5). At the end of each session the application can provide a report of what happened during the game. An xml file, stored on the device, records all data related to a session, such as the time and date when the game was started, the scenarios selected, the type of choice made for each step, and the time span intervening between the moment when the options were shown and when the decision was made. Considering the preliminary level of this study and that the users addressed are children, the usability evaluation has been mainly conducted through beta tests with adult users to assess the proper functioning of the system and to ensure its user-friendliness.

### 2.3. How It Works

The use of AR enables the system to obtain information from the real world triggering an event inside the software application. The physical cube is the means to interact with the application. On each face of the physical cube a sticker is pasted. The stickers represent the markers which are images known by the system and are associated with an ID, as previously highlighted. Each time a marker is recognized, an ID is passed to the software and, according to the running application phase, the information can be used either to set different application parameters or to launch specific events. Figure 3 describes the system flow for each step.

### 2.4. Testing Procedures

#### 2.4.1. Participants

The game has been proposed to 25 children (12 males), aged 4–5 years, attending a kindergarten in Lecco, Italy, at the beginning of the new school-year. The children were selected by the team of the school teachers, according to their own knowledge of the kids, parents’ agreement to participation, organizational factors (e.g., different morning arrival at the school: children who could not be at school in the first observing session were not asked to join the project). Teachers selected children from different sections, mixing them in different groups, in order to compose homogeneous groups for age, level of mutual knowledge (which was generally low since the school year had just started), and multicultural features. There were three children of non-native origin in the group. Indeed, the report of the Italian Ministry of Education on the presence of foreign pupils in the Italian schools in the years 2016–2017 [46] indicates a national figure of 9.2% for kindergarten, highlighting that this figure is higher in the north of Italy and lower in the south. Specifically, the percent of children who are not Italian citizens in kindergartens in the Lombardy region is 16.4%, there were no known disabled children, except for a child with mild mobility difficulties. 

All of the children had had previous experience with electronic devices and at least a PC or a tablet was present in each of the families, as reported by the parents in a questionnaire. The social background of the families was that of a small city, and the school was located in a part of the town where no particular social problems (crime or deviant behaviors) are usually reported. 

The children were divided into three different groups of 8–9 children each and were free to play with the game. The choice to divide the children into three groups allowed to plan game sessions of one hour each, which was deemed to be a reasonable time considering attention times and play skills of children of this age group. Moreover, groups of such size allow rich and dynamic group interactions to be observed, and at the same time are easily managed by adult observers. Their classroom teachers and two trained psychologists followed the activities and prompted the children to interact with the system. All sessions were video-recorded. 

The children’s parents signed informed consent forms as well as permission and release forms for images, videos, and sound recordings. 

#### 2.4.2. Observation Procedures

The timing of the experimentation was organized so as to interfere as little as possible with the kindergarten activities, concentrating the activities in the morning, from school opening to lunch.

A structured observation grid and a questionnaire about the observed behaviors and interactions were filled by the adult participants (teachers and psychologists) at the end of each session. The same grid was filled by two independent observers (trained psychologists) based on offline analysis of the video-recordings.

The grid required evaluation on a Likert five-point scale (from 1 = “not at all” to 5 = “very much”) of a series of variables concerning: “game” (understanding of the game; strategic behaviors; participation), “interactions with peers” (interaction; conflict; cooperation) and “clarification requests” (questions about the device = Q-device; questions about the activities = Q-activity; active help requests = Q-active help). Each variable was rated at three different moments of the playing session: initial, intermediate (after approximately 30 min), and final. Since the variables mainly refer to aspects of interaction and cooperation, the emphasis was on the groups and not on the single individuals, therefore, the ratings for each session are to be referred to the group of children as a whole.

## 3. Results

The test in the kindergarten setting allowed an evaluation of the app functionality.

Eleven filled grids were collected on a set of six total game sessions (three groups, four observers, one observer missing in one session).

First of all, consistency and inter-rater reliability have been analyzed by means of Spearman’s correlations (IBM SPSS Statistics for Windows). All correlation coefficients (rho) among pairs of raters were satisfactory (>0.6), while the overall correlation between the ratings given by participating observers and the ratings based on the analysis of the video-recordings was good, rho(87) = 0.723).

Therefore, the data from different observers were collapsed by averaging, and so were the data on the three groups playing the same kind of activity. The results illustrated in the table are the mean ratings obtained by the groups of children on the different variables at each of the three observation times: T1 (initial), T2 (intermediate) and T3 (final). See Table 1.

**Figure 6:**
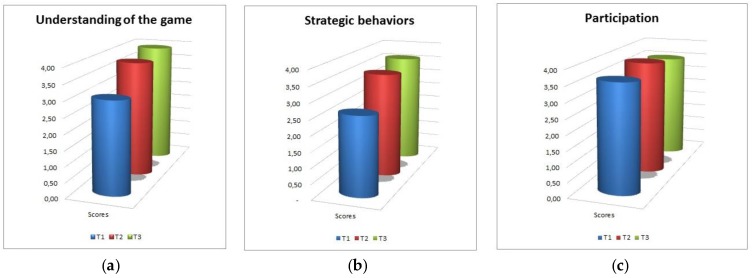
Game-related variables. (**a**): understanding of the game; (**b**): strategic behaviors; (**c**): participation.

As can be seen from Figure 6, understanding of the game improves from the beginning of the session to the end, and achieves good absolute ratings in general. Strategic behaviors in the groups of children tend to increase with time, with both increasing practice and familiarity with the task, especially from T1 to T2. Participation is generally high; it increases from T1 to T2 and remains stable over T3.

**Figure 7:**
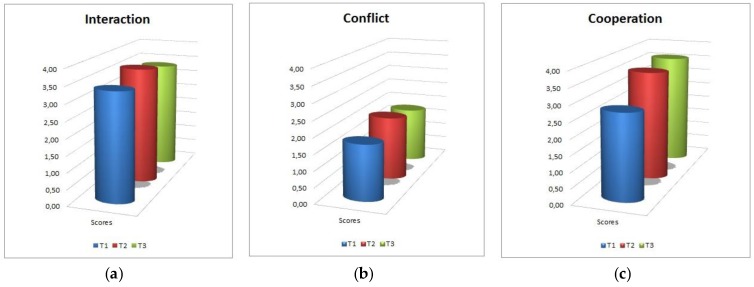
Interaction with peers. (**a**): interaction; (**b**): conflict; (**c**): cooperation.

Variables related to social interaction are represented in Figure 7. Interaction among peers is good and increases over time. Conflict is very low overall, and after a mild increase at mid-session it tends to decrease again to the initial levels. On a more qualitative level, no relevant episodes of conflict among children were observed in any of the sessions and groups.

Cooperation is high and shows an increasing trend.

**Figure 8:**
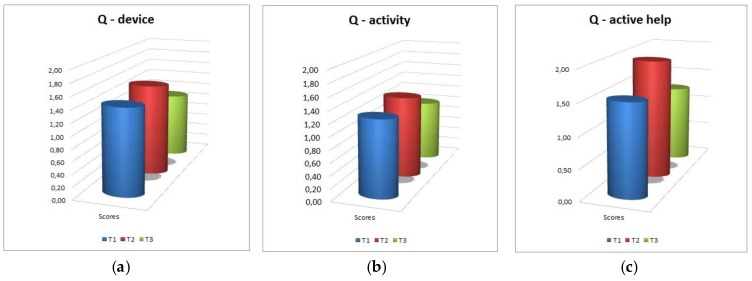
Requests directed to adults and care-givers. (**a**): questions concerning the device; (**b**): questions concerning the activity; (**c**): active help requests.

As to the requests directed to the adults and care-givers, represented in Figure 8, requests of explanations and clarification are infrequent (between 1 = “not at all” and 2 = “rarely”) concerning both the device and the activity, and direct requests of help are quite uncommon.

## 4. Discussion

In the past few years, technology for children has become more and more sophisticated and able to respond to specific needs and purposes. For a long time, it seemed that human–computer interfaces focused on desktop computers. Nonetheless, thanks to technological advancements, novel input devices resting on the user’s interaction with the real non-digital world are constantly increasing their popularity [36]. Tangible user interfaces (TUIs) augment physical objects by coupling them to digital data physical objects and provide users with parallel feedback sources, including passive haptic feedback and digital, visual, or auditory feedback [39].

The present study describes a system intended to stimulate social skills and pragmatic competence. In this work, we describe the design and development of an integrated system which combines the use of smart devices, like tablets or smartphones, a physical cube, the augmented reality (AR) technology, a smart TV, and a software application with educational purposes [1]. The use of AR enables the system to obtain information from the real world triggering an event inside the software application [2].

The system was tested with three groups of normally developing children during kindergarten activities. The results of structured observation reveal that the system is easy to understand, elicits high levels of participation and social interaction, favors strategic behaviors, and can be used by the children with limited need of instruction and support by the adult. Notably, the children’s engagement and their cooperation during the activities improve with familiarization and use of the system.

As it was hypothesized, understanding of the game improves during the session, and achieves a very good level in the end. Additionally, strategic behaviors tend to increase with time. This suggests that accessibility and usability of the app are satisfactory.

Participation is generally high, with an initial increase and then stabilization, while interaction among peers increases over time. This is also in line with the hypothesis.

Conflict is very low overall, in spite of a very mild increase at mid-session. This only partially confirms the expectation about a possible increase in conflict with time, and suggests that, indeed, positive dynamics tied to cooperation and interaction with peers turn out to be more powerful factors than conflictual behaviors. In fact, cooperation is high and steadily increasing, showing that the slight increase of conflictual behaviors along the session is not detrimental to the interaction among the children, but rather seems to depend on a higher level of social exchange.

As to the requests directed to the adults and care-givers, overall requests of explanation, clarification (concerning both the device and the activity), and help are very infrequent and confirm the good levels of accessibility and usability of the system, which appears to be easy to integrate in every day kindergarten activities. Indeed, effective integration of computers in teaching environments depends on the ability of teachers to alter the traditional role of the teacher as a knowledge provider to a conception of the teacher as an organizer, guide, and mediator of computer-assisted learning at all ages, including early childhood [39]. Under such conditions, digital applications have the potential to become important instruments in promoting children’s cognitive and social development, and in improving routine educational work in a kindergarten settings.

The group of children who took part in the experimental activities was well representative of the general pre-school population, with respect to age, gender, and ethnicity, which makes the results easily generalizable. Moreover, its composition allowed the observation of the impact of the application on children with mixed ability levels (several children appeared to have various kinds of difficulties such as hyperactivity, attention disorders, and language or communication disorders, even if not diagnosed—as reported by the teachers and observed by the psychologists who conducted the observation in the school). Nonetheless, the activity was able to involve all the children and to stimulate participation and collaboration independently of individual abilities, and all the children were curious and eager to try and use the application (e.g., taking the cube in their hands and making it interact with the system). Therefore, the potential information emerging from the present observation concerns use with both the typically and the atypically developing children. Indeed, as also stated by [28], there are very few studies on AR applications in the educational setting with special-needs students.

On the whole, then, a positive impact of the activity at both the cognitive and the social level is observed, which constitutes a very encouraging result and suggests that the system can be effectively used in a kindergarten setting to empower communication and problem-solving skills in a cooperative framework. Moreover, the device seems to possess all the pre-requisites to constitute the basis for rehabilitative applications in children with communication disorders, such as Autism Spectrum Disorders and Social (pragmatic) Communication Disorders [23,24]. In fact, its characteristics incorporate all the elements that have been shown to produce positive effects in children with social and communication problems: it greatly exploits nonverbal communication, provides funny, emotionally expressive characters, and provides examples of simple and concrete daily-life situations that constitute problematic situations for the children, illustrating social rules and providing suggestions to face and solve the difficulties. Emotionally-expressive avatars are one of the most interesting options of new educating systems. Results of surveys among educators of autistic children suggest that not only do most of the children recognize the avatars’ emotions, but also that the avatar’s emotional state advances the educational process [47,48]. Training studies have further suggested that children with autism show greater improvements in emotion recognition when programs use cartoons rather than photographs of real faces [49].

The users, both typically and atypically developing, will thus be engaged in social activities without experiencing the anxiety-loaded constraints of real-life interactions, giving them time and guidance to decode and analyze all the elements of the situation, to decide the goal of the response, anticipate the consequences for each possible answer, and decide which answer may be the most fruitful and most rewarding one. The basic idea is that they will form habits for analysis and evaluation of social situations that will help them choose better strategies in real-life problematic situations. Moreover, the positive feelings evoked by the solution of the problem-solving situations should help them build progressively greater self-esteem and self-confidence, which, in a sort of virtuous circle, should reduce their spontaneous resistance in engaging in real-life interactions.

So-called stealth education, which presents entertaining features of the learning situation in a seamless, game-like manner, provides an important method to allow children to explore and learn from materials and it can provide experiences that arouse emotions, enhancing the significance of the experience itself, and the probability that such experience will be remembered [30]. A similar study focusing on storytelling through an interactive robot [31] promoted children’s emotional involvement in the learning process and facilitated an increase in on-task behaviors and at the same time a reduction in challenging behaviors.

Considering Gee’s principles [9], it can be shown that such activities possess all the requisites for fostering active learning through VR-based games. In particular, the app described in the present study is able to fully represent the “active, critical learning principle” requiring that the learning environment encourage active and critical learning; the “design principle” placing learning at the core of the learning experience; and the “psychosocial moratorium principle”, stating that learners can take risks in a space where real-world consequences are lowered.

As stated before, the present study focuses on the effects of such stimulation at the group level, before extending the investigation of the effects on typically developing children (or even more desirable, on the general preschool population) at the individual level. The results are very encouraging and suggest that the app can be successfully employed for empowerment of social abilities in group activities. Further studies are envisaged, testing the impact of the app on children with neurodevelopmental disorders, especially those involving deficits of executive functions, problem-solving skills and social abilities and communication skills or pragmatic competence such as children with ASD [24], pre-term born children [50], or other vulnerable populations, including children from immigrant families, ethnic minorities, or low SES contexts [51]. This could prove very useful both as an empowerment tool, and as an instrument to foster greater inclusion and prevent isolation, aggression, or bullying behaviors [52].

## 5. Conclusions

The results of the study show that an integrated system, such as the one described above, incorporating aspects of tangible user interfaces and designed within the framework of technology-enhanced learning and, more generally, of neuroconstructivism, can have positive effects on social interaction in a kindergarten group context, arising long-lasting interest and active co-operation in the children. Such a system, specifically aiming to stimulate pragmatic skills and general problem-solving abilities in preschoolers, regardless of their starting abilities, can be a model for other AR-based applications for cognitive empowerment in typically and atypically developing children, not only pursuing cognitive improvement, but also fostering inclusion and social integration, as would be desirable in both educational and group rehabilitation settings.

## Figures and Tables

**Table 1:** Variables scores at each of the three observation times: T1 (initial), T2 (intermediate), and T3 (final).

Variables	Time		
	T1	T2	T3
Understanding of the game	3.00	3.75	3.92
Strategic behaviors	2.58	3.42	3.58
Participation	3.58	3.75	3.50
Interaction	3.33	3.58	3.33
Conflict	1.75	2.00	1.75
Cooperation	2.75	3.50	3.58
Q-device	1.42	1.50	1.08
Q-activity	1.25	1.33	1.00
Q-active help	1.50	1.92	1.25

## Supplementary Materials

The following are available online at http://www.mdpi.com/1424-8220/18/7/2368/s1, Video S1: Part of the “Playground” scenario of Giok the Alien’s story.
